# Self-Renewal and Differentiation of Adipose-Derived Stem Cells (ADSCs) Stimulated by Multi-Axial Tensile Strain in a Pneumatic Microdevice

**DOI:** 10.3390/mi9110607

**Published:** 2018-11-19

**Authors:** Chih-Hao Chiu, Yun-Wen Tong, Wen-Ling Yeh, Kin Fong Lei, Alvin Chao-Yu Chen

**Affiliations:** 1 Department of Orthopedic Surgery, Chang Gung Memorial Hospital, Taoyuan 333, Taiwan; joechiu0115@gmail.com (C.-H.C.); a0925205872@gmail.com (Y.-W.T.); 2 Bone and Joint Research Center, Chang Gung Memorial Hospital, Linkou 333, Taiwan; yeh610128@gmail.com; 3 Department of Orthopedic Surgery, Chang Gung Memorial Hospital, Linkou 333, Taiwan; 4 Graduate Institute of Biomedical Engineering, Chang Gung University, Taoyuan 333, Taiwan; 5 Department of Radiation Oncology, Chang Gung Memorial Hospital, Linkou 333, Taiwan

**Keywords:** microdevice, tensile stimulation, adipose-derived stem cells, self-renewal, differentiation

## Abstract

Adipose-derived stem cells (ADSCs) were suggested for treating degenerative osteoarthritis, suppressing inflammatory responses, and repairing damaged soft tissues. Moreover, the ADSCs have the potential to undergo self-renewal and differentiate into bone, tendon, cartilage, and ligament. Recently, investigation of the self-renewal and differentiation of the ADSCs has become an attractive area. In this work, a pneumatic microdevice has been developed to study the gene expression of the ADSCs after the stimulation of multi-axial tensile strain. The ADSCs were cultured on the microdevice and experienced multi-axial tensile strain during a three-day culture course. Self-renewal and differentiation abilities were investigated by mRNA expressions of NANOG, sex determining region Y-box 2 (SOX2), octamer-binding transcription factor 4 (OCT4), sex determining region Y-box9 (SOX9), peroxisome proliferator-activated receptor gamma (PPAR-γ), and runt-related transcription factor 2 (RUNX2). The result showed that the genes related self-renewal were significantly up-regulated after the tensile stimulation. Higher proliferation ratio of the ADSCs was also shown by cell viability assay. The microdevice provides a promising platform for cell-based study under mechanical tensile stimulation.

## 1. Introduction

Tendon and ligament injuries induce serious consequences, such as debilitating pain and reduced joint function. Rotator cuff repairs are conducted over 250,000 times in North American each year. High re-tear rates still occur in spite of newly developed techniques being adopted to increase the repair strength [1,2,3,4]. Thus, numerous methods had been proposed to enhance tendon healing, including stem cells, mechanical forces, pulsed electromagnetic fields (PEMFs), drugs, gene therapy, osteoconductive materials, cell-based therapy, biodegradable scaffolds, low-intensity pulsed ultrasound treatment, and biomimetic patches [4,5,6,7,8,9,10,11,12]. The use of human stem cells is one of the more popular research approaches for soft tissue healing. In the past decade, adipose-derived stem cells (ADSCs) were suggested to be an adult stem cell population isolated from adipose tissue [13,14]. The largest study using adipose-derived stromal vascular fraction cells (SVF cells) to treat osteoarthritis involved 1128 patients [15]. Most cases consistently reported that adipose-derived cell therapy for treating degenerative osteoarthritis is safe and effective. Moreover, some reports revealed the ADSCs contain unique populations of cells that suppress the inflammatory responses, and thus may further contribute to tissue regeneration and repair of damaged tendon tissues [16,17]. A review article surmised ADSCs could be used for the regeneration of damaged tissues [18]. In addition, because stem cells are known to be multipotent, nonhematopoietic stromal cells that have the potential to undergo self-renewal, they have the ability to differentiate into bone, tendon, cartilage, and ligament [19,20,21,22].

In the literature, much of the work has focused on human bone marrow mesenchymal stem cells (MSCs). Mechanical stretching was shown to induce the proliferation and differentiation of MSCs into tenocytes associated with cumulative elongation [23,24]. MSC-to-tenocyte under 3–10% mechanical stretching at 1 Hz was evaluated by analyzing mRNA expression levels [25,26]. Studies have shown that applying intermittent mechanical tension to MSCs (every few hours, several times a day, for several days) promotes osteogenic differentiation, whereas applying continuous mechanical tension to MSCs inhibits osteogenic differentiation. On the other hand, osteogenic differentiation of the ADSCs was reported to be induced under uniaxial stretching [27,28,29]. Cyclic tensile strain significantly increased gene expressions of bone morphogenetic protein 2 (BMP2) and runt-related transcription factor 2 (RUNX2), which responded to the modulation of the osteogenic differentiation. Also, cyclic tensile strain led to more aligned and organized ADSCs. Moreover, a study reported the comparison of ADSCs and MSCs under mechanical stretching [30]. The results concluded that ADSCs are more rapid responders to mechanical stretching and have greater potential than MSCs in osteogenesis. Thus, more and more works have been focused on the investigation of the proliferation and differentiation of ADSCs stimulated by mechanical stretching.

In the above in vitro experiments, uniaxial stretching apparatus were used to apply single directional strain to the cells [23,24,25,26,27,28,29,30,31,32,33,34]. However, during the movement of the body, the soft tissues are subjected to multi-axial mechanical strain. Development of multi-axial mechanical stretching microdevice is necessary to mimic the native in vivo environment. With the mature development of microfabrication technology, a number of microdevices have been developed for various biomedical and clinical applications [35,36,37,38,39]. The microdevices are often interpreted as miniaturized versions of a conventional laboratory. Pioneering work in this domain has demonstrated the construction of a complicated system integrating on-off valves, switching valves, and pumps [40,41]. Then, various other biomedical applications have been demonstrated, including DNA diagnostics, immunoassay, and cell-based assays. For example, an electrokinetically controlled DNA hybridization microdevice was developed and consisted of an H-type channel structure [42]. The microdevice could conduct all processes, from sample dispensing to hybridization detection, within 5 min. Moreover, a microdevice for immunoassay was developed for automatically pumping sample and reagents into respective reaction chambers [43]. The micropumps actuated by a pneumatic mechanism could manipulate reagents for the detection of hepatitis C virus and syphilis from serum samples. Furthermore, biological cells have been shown to be cultured in a controlled microenvironment for investigating their physiology and biochemistry under the tested conditions [44,45]. A microfluidic device composed of 10 × 10 culture chambers was demonstrated in a high throughput cell-based screening application. Mammalian HeLa cells were cultured in the chamber and proliferated nearly to confluency after 7.5 days to show promising evidence of a microfluidic cell culture model. In these microfluidic devices, one of the commonly used materials is polydimethylsiloxane (PDMS), which is optically transparent, flexible, and bio-compatible. Thus, a PDMS deflective membrane could be developed to provide compressive force for cell stimulation [46]. Cell viability was investigated under different levels of compressive force. Some reports demonstrated the ability to culture cells on the flexible membrane providing tensile stress [47,48,49,50]. The cyclic tensile stress could be generated and stimulated MSCs to promote proliferation, osteogenesis, and reduced adipogenesis [47]. Nowadays, the PDMS membrane-type microdevices fabricated by microfabrication technology are recognized to be a promising tool for cell-based assays.

In the current work, a PDMS-based microdevice was developed for providing multi-axial mechanical tensile stretching for cell stimulation. The device was composed of nine culture chambers with a pneumatic mechanism. For each chamber, the bottom surface was a deflective membrane actuated by compressed air. The pressure and actuation frequency were controlled by an in-house built instrument comprised of a compressed air pump, a pressure gauge, and an electronic valve controlled by a microcontroller. ADSCs were cultured in the chamber and experienced multi-axial tensile strain during a three-day culture course. Self-renewal ability was investigated by mRNA expressions of NANOG, SOX2, and OCT4. Moreover, the chondrogenic gene SOX9, the adipogenic gene PPAR-γ, and the osteogenic gene RUNX2 were analyzed to study the cell differentiation. The result showed that NANOG, SOX2, and OCT4 were significantly up-regulated after the tensile stimulation. A higher proliferation ratio of the ADSCs was also shown by the cell viability assay. This indicated self-renewal of the ADSCs was confirmed after the mechanical stretching.

## 2. Materials and Methods

### 2.1. Harvest and Isolation of Human ADSCs

**Figure 1:**
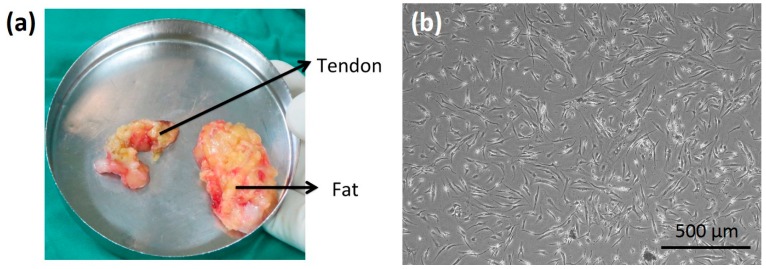
(**a**) Photograph of the harvested fat tissue. (**b**) Microscopic image of the first passage of adipose-derived stem cells (ADSCs).

**Figure 2:**
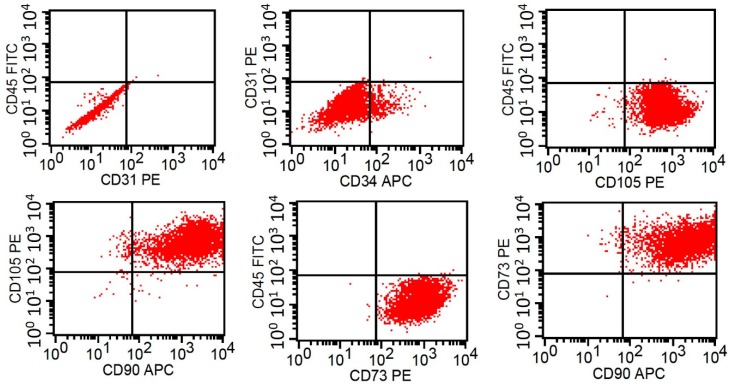
Analysis of the surface markers of ADSCs by using flow cytometry.

Human ADSCs were isolated from discarded tissue removed during total knee arthroplasty. The approval of the tissue collection was given by the Institutional Review Board at Chang Gung Memorial Hospital, Linkou, Taiwan (IRB No. 2016014923). Seven patients were recruited for this study, and their age and gender are listed in Table 1. Before the surgery, the patients had been given informed consent. The harvested fat tissue is shown in Figure 1a. The tissue was digested in an enzymatic solution containing 300 U/mL collagenase Type II (Gibco, Invitrogen, Paisley, UK) and cultured at 37 °C for 2 h. After the digestion process, the solution was filtered and centrifuged at 1200 rpm for 5 min at room temperature to separate floating mature adipocytes. The cell pellet was then suspended in culture medium (RPMI 1640 supplemented with 10% fetal bovine serum and 1% antibiotics) and maintained in standard culture plates. The microscopic image of the first passage of ADSCs is shown in Figure 1b. The ADSCs were confirmed by analyzing the surface markers using flow cytometry, as shown in Figure 2. The result showed that over 90% of the isolated cells express CD90+/CD73+/CD105+/CD31−/CD34−/CD45−. This indicated the cells had the phenotypic and functional features of stem cells [21,22]. The ADSCs above five passages were discarded because of the possibility of phenotypic drift.

### 2.2. Fabrication of the Microdevice Providing Multi-Axial Tensile Strain

**Figure 3:**
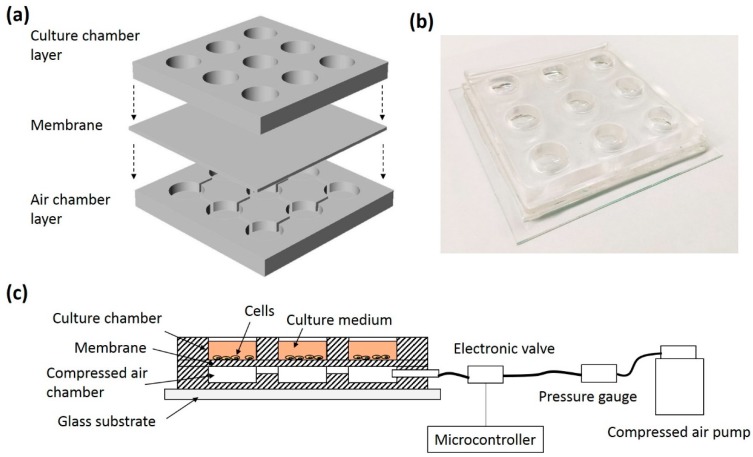
(**a**) Illustration of the design of the microdevice. (**b**) Photograph of the microdevice. (**c**) Illustration of the experimental setup of the cells stimulated by the multi-axial tensile strain.

A PDMS-based microdevice was developed for culturing ADSCs and providing multi-axial tensile strain to the cells. An illustration and photograph of the microdevice are respectively shown in Figure 3a,b. The device consisted of 3 PDMS layers, including a culture chamber layer, a membrane, and an air chamber layer. The culture chamber layer was a 5 mm thick PDMS layer with a 3 × 3 array of circular through holes with a diameter of 10 mm. The membrane was a 100 μm thick PDMS layer. The air chamber layer was composed of a 3 × 3 array of chambers (1 mm in height and 10 mm in diameter) connected with channels. The membrane and the air chamber layer were fabricated by soft lithography. Briefly, poly(methyl methacrylate) (PMMA) molds were respectively fabricated by a micro-engraving machine (EGX-400; Roland Corporation, Hamamatsu, Japan). Afterward, PDMS pre-polymer and curing agent (Sylgard^®^ 184; Dow Corning, Midland, MI, USA) in (*w*/*w*) 10:1 were manually mixed and degassed in a vacuum chamber. Then, the mixture was poured into the PMMA molds and solidified in an oven at 70 °C for 1 h. Subsequently, the PDMS layers were respectively peeled off from the molds. Three PDMS layers were bonded by an oxygen plasma (PDC-32G; Harrick Plasma, Ithaca, NY, USA) and placed on a glass substrate for solid support. Therefore, the microdevice with a 3 × 3 array of culture chambers was fabricated. The bottom surface of each chamber was a deflective membrane, which can be actuated by the pressure change of the air chamber. The pressure and actuation frequency were controlled by an in-house built instrument comprised of a compressed air pump, a pressure gauge, and an electronic valve controlled by a microcontroller. ADSCs were cultured in the chamber and experienced multi-axial tensile strain during the culture course. An illustration of the experimental setup is shown in Figure 3c.

### 2.3. ADSCs Cultured on the Microdevice

Before the cell culture experiment, the microdevice was sterilized under ultraviolet light overnight. In order to improve cell adhesion on the PDMS surface, 50 μL collagen solution in 50 μg/mL was respectively added to each culture chamber and stored at 4 °C overnight. Then, the culture chambers were washed and ready for the cell culture experiment. One hundred thousand (10^5^) cells were added to each chamber and cultured in a 37 °C and 5% CO_2_ humidified incubator (370; Thermoscientific, Waltham, MA, USA) overnight. The cells were seeded and spread on the bottom surface, i.e., membrane, of the chamber. After cell stabilization, multi-axial tensile strain at 1 Hz was applied to the cells by applying 5 kPa compressed air to the air chambers. The cells were continuously stimulated for the following 1 or 3 days. Subsequently, the cells were harvested after the culture course, and the mRNA expression level was examined. Relative gene expression level was defined as the gene expression level of cells with stretching stimulation divided by the gene expression level of cells without stretching stimulation (control). The change of the gene expression was investigated to evaluate the properties of self-renewal and differentiation.

The deformation of the membrane was quantified by images captured from a microscope installed horizontally. From the images, the height and angle of the deformed membranes were measured using ImageJ computer software (Version Java 1.8.0, National Institutes of Health (NIH), Bethesda, MD, USA). Then, the contour of the deformed membrane, *f*(*x*), could be estimated by quadratic equation fitting. The axial elongation, *x*, was calculated by

(1) $$x=\int_{b}^{a}\sqrt{1+f\left(x\right){}^{\prime }}dx$$

Thus, the deformation of the membrane could be controlled by the applied pressure. The cells cultured on the membrane received the multi-axial tensile strain during the culture course.

### 2.4. Investigation of mRNA Expressions

After the culture course, the cells were harvested and mRNA expression was then investigated by using real time polymerase chain reaction (PCR). Briefly, the total RNA of the cells was extracted using a GENEzol^TM^ TriRNA Pure Kit (GZX100; Geneaid, New Taipei City, Taiwan) according to the supplier’s instruction. Then, cDNA was synthesized by a cDNA synthesis kit (18080-400; Invitrogen, Waltham, MA, USA) using a T100^TM^ Thermal Cycler (Bio-Rad Laboratories, Inc., Hercules, CA, USA). The relative quantity of mRNA was determined by a CFX Connect^TM^ Real-time PCR Detection System (Bio-Rad, USA) using TaqMan™ Universal Master Mix II, with UNG (4440038; ThermoFisher Scientific, Waltham, MA, USA). The mRNA expressions of NANOG, SOX2, OCT4, SOX9, PPAR-γ, and RUNX2 were examined. The TaqMan^®^ gene expression assays were used and are listed in Table 2. The expression of glyceraldehyde 3-phosphate dehydrogenase (GAPDH) was used as the internal control. Relative gene expression level was used to analyze the change of gene expression of cells with and without tensile strain. It was defined as the fold of gene expression of cells with and without tensile strain. Thus, the control (without tensile strain) is “1”.

### 2.5. Quantification of Cell Proliferation

The number of living cells was quantified by bio-assay, such as a WST-1 assay (Roche Applied Science, Indianapolis, IN, USA). After the culture course, the culture medium was removed, and the reagent of the WST-1 assay in a dilution of 1:10 (*v*/*v*) was added to each chamber. The reagent reacted with the respiratory chain of mitochondria, and the color intensity of the reagent changed according to the reaction level. After incubation at 37 °C for 2 h, the reacted reagent was collected and quantified by a microplate reader (ELx800; BioTek Instruments, Winooski, VT, USA) at an absorbance of 440 nm with a reference wavelength of 660 nm. Thus, the color intensity of the reacted reagent could be represented by optical density (OD). The proliferation ratio was defined as the OD value at the end of the culture course divided by the OD value at the beginning of the culture course.

## 3. Results and Discussion

### 3.1. Investigation of the Membrane Deformation

**Figure 4:**
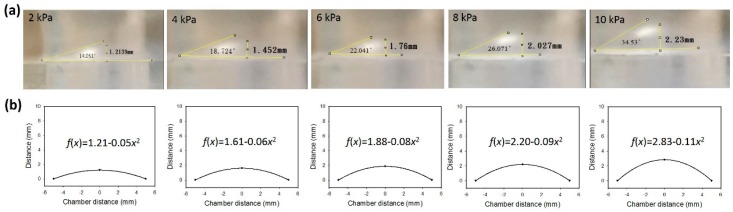
(**a**) Side view photographs of the deformed membrane under different applied pressures. (**b**) Contours of the deformed membrane fitted by quadratic equation.

**Figure 5:**
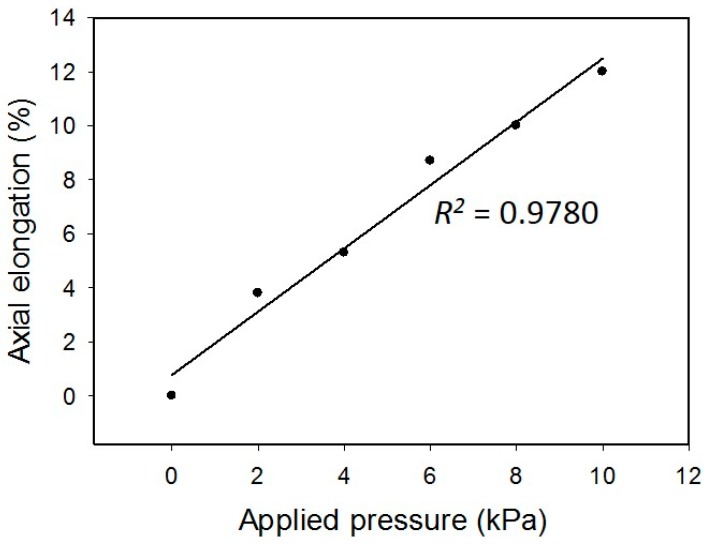
Correlation between the applied pressure and the axial strain.

The deformation of the membrane was induced by the pressure applied to the air chamber. The pressures of 2, 4, 6, 8, and 10 kPa were regulated to induce different levels of deformation. Side view photographs of the deformed membrane were captured under different applied pressures and are shown in Figure 4a. The height and angle of the deformed membranes are also indicated in the photographs. Obviously, higher pressure generated larger deformation. Hence, the contours of the deformed membrane were fitted by quadratic equation. The results are shown in Figure 4b. The axial elongation and strain were calculated and are listed in Table 3. The correlation between the applied pressure and the axial strain is shown in Figure 5. A linear correlation with an R-squared value of 0.9780 was obtained. This indicates the deformation of the membrane was in the elastic region. In addition, an axial strain ranging from 0 to 12% was achieved, and that is suitable to the mechanical stretching study for cells.

### 3.2. mRNA Expressions of Cells after Mechanical Stretching Stimulation

In this study, self-renewal genes (NANOG, SOX2, and OCT4), a chondrogenic gene (SOX9), an adipogenic gene (PPAR-γ), and osteogenic genes (RUNX2) were examined to study the self-renewal and differentiation capacity of the ADSCs after mechanical stretching stimulation. NANOG is a transcription factor in embryonic stem cells and functions with SOX2 and OCT4 to maintain pluripotency [51,52]. Thus, self-renewal capacity could be analyzed by NANOG, SOX2, and OCT4. Moreover, transcription factor SOX9 has an essential role during chondrocyte differentiation [53]. PPAR-γ is a member of the nuclear hormone receptor superfamily and has a key role in adipose cell differentiation [54]. RUNX2 was reported to promote osteogenic differentiation [55]. Thus, the multipotential differentiation of the ADSCs was studied by analyzing SOX9, PPAR-γ, and RUNX2.

**Figure 6:**
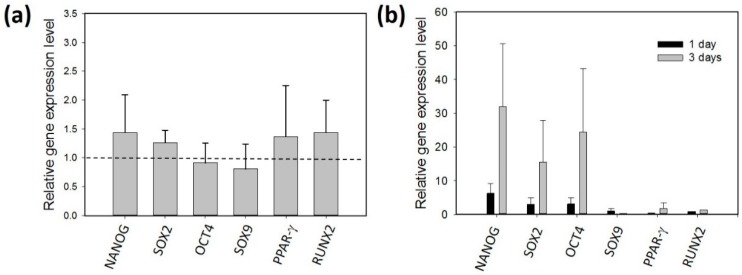
(**a**) Control experiment that cultured the ADSCs without mechanical stretching stimulation. The gene expression level was compared before and after a three-day culture course. (**b**) Investigation of the gene expression of the ADSCs after the stimulation of the multi-axial tensile strain. The ADSCs were respectively stimulated by the multi-axial tensile strain for one day and three days. The ADSCs were isolated from the tissue samples of patients #1 and #2. The data were generated from three repeated experiments for each tissue sample. The data are presented as mean ± standard error.

A control experiment (without mechanical stretching stimulation) was conducted to investigate the gene expression level of the ADSCs. The ADSCs (isolated from the tissue samples of patients #1 and #2) were cultured in the culture chambers for three days. The gene expression level was compared before and after the three-day culture course. Figure 6a reveals the gene expression was not changed. This indicates the culture environment did not induce the change in the gene expression of the cells. Then, the gene expression of the ADSCs after the stimulation of the multi-axial tensile strain was investigated. The ADSCs (isolated from the tissue samples of patients #1 and #2) were respectively stimulated by the multi-axial tensile strain for one day and three days. The result is shown in Figure 6b. This revealed NANOG, SOX2, and OCT4 were up-regulated after the stimulation. The result implies that the self-renewal capacity of the ADSCs was induced by mechanical stretching. Importantly, the cells stimulated for three days had a higher influence than those for one day. On the other hand, the gene expressions of SOX9, PPAR-γ, and RUNX2 were not changed, and this implies the differentiation of the ADSCs was not induced.

**Figure 7:**
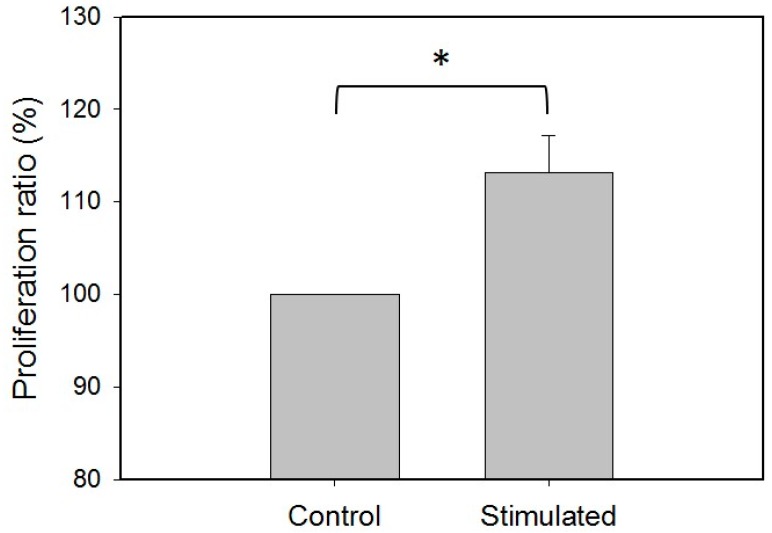
Investigation of the cell proliferation after the stimulation of the multi-axial tensile strain for three days. The control group represents the cells that were cultured without tensile strain. The stimulated group represents the cells were cultured with tensile strain. The ADSCs were isolated from the tissue samples of patients #1 and #2. The data were generated from three repeated experiments for each tissue sample. The data are presented as mean ± standard error. The results were analyzed using one-way analysis of variance (ANOVA). Statistical significance is indicated as * for *p* < 0.05.

**Figure 8:**
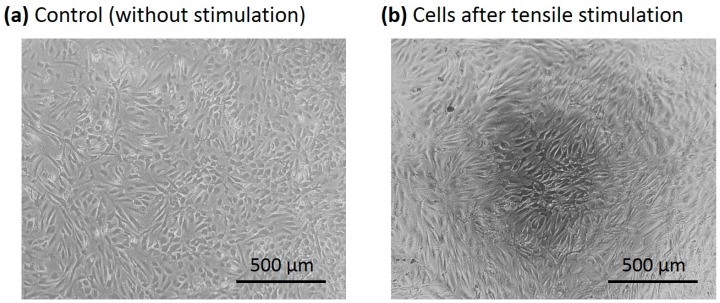
Microscopic images of the ADSCs. (**a**) Control group (without stimulation). (**b**) The cells stimulated by multi-axial tensile strain for three days.

Because the self-renewal capacity of the ADSCs could be induced by mechanical stretching, investigation of the cell proliferation was conducted. The ADSCs (isolated from the tissue samples of patients #1 and #2) were stimulated by the multi-axial tensile strain for three days. The cell proliferation ratio was quantified and compared with the control group (the cells cultured without stimulation for three days). Figure 7 shows that the proliferation ratio of the stimulated cells was increased. That indicates the mechanical stretching could also enhance cell proliferation. Moreover, microscopic images of the cells with/without stimulation were captured and are shown in Figure 8. The images show the alignment of the stimulated ADSCs was clearly different from the control group.

### 3.3. Gene Expression Influenced by Individuals

**Figure 9:**
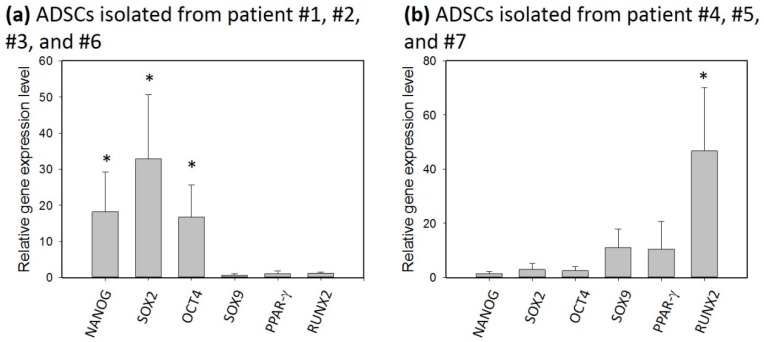
Investigation of the gene expression of the ADSCs after the stimulation of the multi-axial tensile strain. (**a**) The ADSCs were isolated from patients #1, #2, #3, and #6. (**b**) The ADSCs were isolated from patients #4, #5, and #7. The ADSCs were respectively stimulated by the multi-axial tensile strain for three days. The data were generated from three repeated experiments for each tissue sample. The data are presented as mean ± standard error. The results were analyzed using one-way analysis of variance (ANOVA). Statistical significance was compared to the control and is indicated as * for *p* < 0.05.

In the previous section, the self-renewal capacity of the ADSCs was shown to be induced by the mechanical stretching. However, individual variability is known to be one of the issues of the influence of gene expression. The individual variability might be due to a patent’s gender and age. Also, since the tissues were harvested during total knee arthroplasty surgeries, the tissues may have different levels of damage. Thus, more patients were recruited in order to study the individual variability. The ADSCs were respectively stimulated by the multi-axial tensile strain for three days, and the relative gene expression levels were investigated, as shown in Figure 9. Distinct outcomes were revealed from the figure. For the ADSCs isolated from patients #1, #2, #3, and #6, the genes related to self-renewal capacity (NANOG, SOX2, and OCT4) were up-regulated, and other genes related to differentiation (SOX9, PPAR-γ, and RUNX2) were not changed. In contrast, for the ADSCs isolated from patients #4, #5, and #7, the genes related to differentiation (SOX9, PPAR-γ, and RUNX2) were up-regulated, especially for RUNX2. This implies osteogenic differentiation was promoted. This agrees with the results from the literature [27,28,29]. The result shows individual variability highly dominates the outcome of the gene expression after the stimulation.

## 4. Conclusions

A PDMS-based microdevice has been developed to study the gene expression of ADSCs after the stimulation of multi-axial tensile strain. The results indicated that the gene expressions of NANOG, SOX2, and OCT4 were up-regulated, while SOX9, PPAR-γ, and RUNX2 were not changed after the stimulation. Importantly, the cells stimulated for three days had a higher influence than those for one day. Also, proliferation of the ADSCs was increased by the mechanical stretching. This implied the self-renewal capacity of the ADSCs was shown to be induced. However, the result was highly influenced by the individual variability. Another set of the ADSCs isolated from the other patients showed osteogenic differentiation was promoted after the stimulation. Nevertheless, the microdevice provides a promising platform for the study of ADSCs stimulated by multi-axial tensile strain.

## Figures and Tables

**Table 1:** Summary of the age and gender of the patients.

Patient Number	Age	Gender
1	83	Female
2	77	Male
3	69	Female
4	78	Male
5	78	Female
6	63	Female
7	70	Female

**Table 2:** TaqMan^®^ gene expression assays.

Gene	Assay Number (ThermoFisher Scientific, Waltham, MA, USA)
GAPDH	Hs03929097_g1
NANOG	Hs02387400_g1
SOX2	Hs00602736_s1
OCT4	Hs01895061_u1
SOX9	Hs00165814_m1
PPAR-γ	Hs01115513_m1
RUNX2	Hs00231692_m1

**Table 3:** The elongation and strain of the membrane induced by different pressures.

Applied Pressure (kPa)	Axial Elongation (mm)	Axial Strain (%)
0	10.00	0
2	10.38	3.8
4	10.53	5.3
6	10.87	8.7
8	11.00	10
10	11.20	12
